# Stochastic Reaction–Diffusion Model of the Binding of Monoclonal Antibodies to CD4 Receptors on the Surface of T Cells

**DOI:** 10.3390/ijms21176086

**Published:** 2020-08-24

**Authors:** Lili Wang, Paul C. DeRose, Sarah L. Inwood, Adolfas K. Gaigalas

**Affiliations:** 1 National Institute of Standards and Technology, Gaithersburg, MD 20899, USA; lili.wang@nist.gov (L.W.); paul.derose@gmail.com (P.C.D.); sarah.inwood@nist.gov (S.L.I.); 2 Fluorescence Spectroscopy Consultant, 2650 Lake Shore Drive, Riviera Beach, FL 33404, USA

**Keywords:** stochastic simulation, CD4^+^ T cells, labeling reaction, monovalent, bivalent, CV%, flow cytometer, antibodies bound per cell

## Abstract

A stochastic reaction–diffusion model was developed to describe the binding of labeled monoclonal antibodies (mAbs) to CD4 receptors on the surface of T cells. The mAbs diffused to, adsorbed on, and underwent monovalent and bivalent binding to CD4 receptors on the cell surface. The model predicted the time-dependent nature of all populations involved in the labeling process. At large time, the populations reached equilibrium values, giving the number of antibodies bound to the T cell (ABC) defined as the sum of monovalently and bivalently bound mAbs. The predicted coefficient of variation (CV%) of the (ABC) values translated directly to a corresponding CV% of the measured mean fluorescence intensity (MFI). The predicted CV% was about 0.2% from the intrinsic fluctuations of the stochastic reaction process, about 5% after inclusion of the known fluctuations in the number of available CD4 receptors, and about 11% when fluctuations in bivalent binding affinity were included. The fluorescence detection process is expected to contribute approximately 7%. The abovementioned contributions to CV% sum up to approximately 13%. Work is underway to reconcile the predicted values and the measured values of 17% to 22%.

## 1. Introduction

Flow cytometers measure mean fluorescence intensity (MFI) emitted from cells with antibodies that contain conjugated fluorophores. The antibodies bind to specific receptors on the surface of the cell, and, with proper calibration, the MFI signal can yield the number of antibodies bound to the cell (ABC) [1]. Another property of the observed fluorescence signals is the coefficient of variation (CV), defined as the standard deviation (SD) of the fluorescence signals divided by the MFI. The CV has not been used to obtain additional information about the cell. This work attempts to provide a basis for interpreting the CV in terms of relevant biological information such as variety of CD4^+^ T-cell subtypes present in a constantly adapting immune system.

Motivated by the many possible sources of signal fluctuations, this work utilizes a stochastic reaction-diffusion model to guide the estimate of possible fluctuations and, thus, guide the interpretation of the CV. Uncertainty due to instrument factors such as electronic gain is decreased because the CV is a ratio based on the same set of fluorescence measurements. In keeping with tradition to represent the SD as a percentage of the MFI, the CV is multiplied by 100 and denoted by CV%. T cells are an important component of the immune system [2] and play an important role in new immunotherapy [3]. Recently, T cells have also become prime candidates for standardizing quantitative flow cytometer measurements [4]. This is primarily because the number of CD4 receptors on the surface of T cells is reasonably constant for all normal people [5], and thus can serve as convenient standard for quantification of flow cytometer measurements. Characterizing CD4^+^ T cells is critical for the development of flow cytometer standards. Of special concern is the variability of the process of labeling T cells with monoclonal antibodies (mAbs) for the CD4 receptor. This manuscript describes a model for the labeling process of T cells. The model starts with a description of the initial step during which the mAbs in the labeling suspension are transported to the vicinity of the cell surface, where the mAbs interact with adsorption sites on the surface of the cell. The model then describes the binding reaction between the adsorbed mAbs and the receptors (Rs) on the cell surface. The analysis suggests that the fluctuations in the number of mAb receptors on the T cell contribute about 5% to the CV% of MFI. Including fluctuations in the bivalent binding constant, k_bp_, this leads to a larger CV% of 11%. Fluorescence detection fluctuations add another 7% for a CV% value of 13%. This is less than the observed CV% of 17% to 22%.

## 2. Results

### 2.1. Measurement of CV% of T Cells Labeled with mAb Fluorophore Conjugates

**Figure 1:**
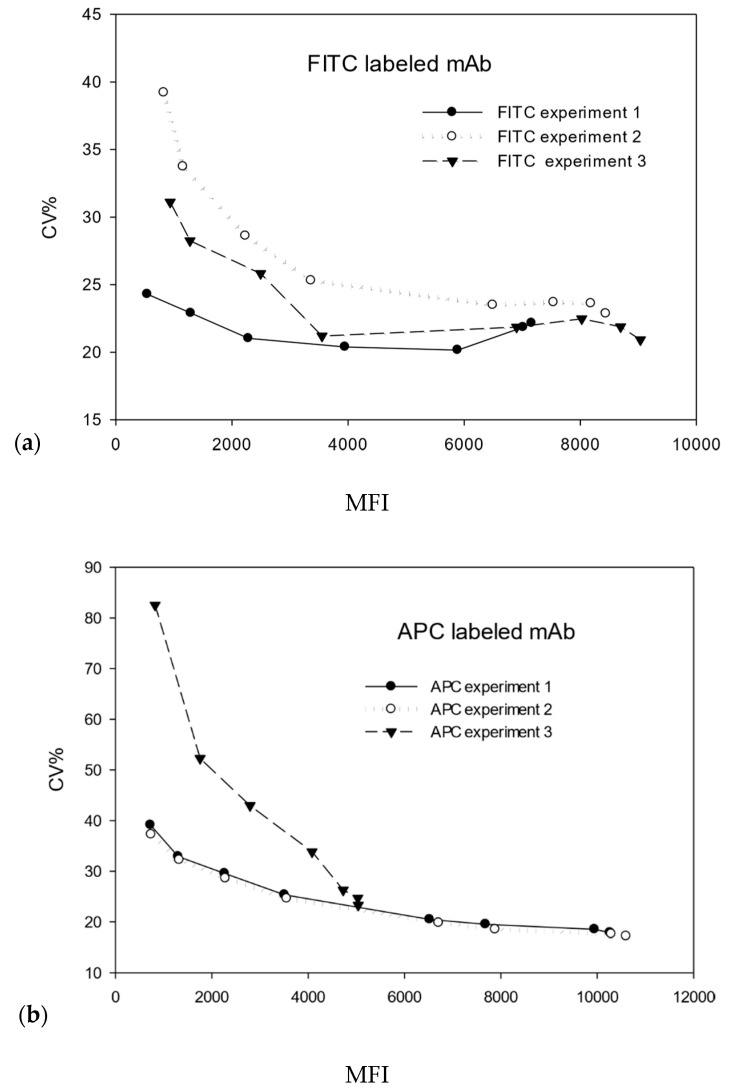
Dependence of CV% on mean fluorescence intensity (MFI) for peripheral blood mononuclear cells (PBMCs) labeled with fluorescein isothiocyanate (FITC) and allophycocyanin (APC). Graphs (**a**,**b**) show results for three repetitions of the same series of measurements for each label. The large variation in FITC-CV% at small MFI is most likely due to cell settling in the labeling solution. The CV% approaches a reproducible value at large values of MFI, where the monoclonal antibody (mAb) concentration in the labeling solution is much higher.

The model presented below describes the time evolution of the number of mAbs bound on the surface of cells during the incubation period. Sufficient elapsed time was allowed in the model, to ensure that a binding equilibrium was reached. The results of the stochastic simulation were compared to the measurements of CV% of MFI shown in Figure 1, which gives CV% as a function of label load on the T cells, as given by MFI. Figure 1a,b represents CV% measurements for mAb conjugated with fluorescein isothiocyanate (FITC) and allophycocyanin (APC), respectively. The data in Figure 1a,b show a dependence of CV% on MFI for three independent measurements for each label. Clearly the curves in Figure 1a,b are similar but different in detail. The values of CV% at low MFI were all different in the case of FITC label and merged to a value of 22 ± 1 at large MFI. Two of the curves (open and full circles) in Figure 1b were similar, while the curve with triangle symbols deviated significantly from the pair. It is important to note that all three curves in Figure 1b merged to a CV% value of 17 ± 1 at large values of MFI. The common CV% value at large MFI suggests that, at saturating label concentrations, the measured CV% is representative of the T-cell population.

The lack of reproducibility in the CV% vs. MFI dependence at low values of MFI is troubling and is clearly due to uncontrolled factors. Most likely the dependence of CV% shown in Figure 1a,b is dominated by fluctuations in the mass transport during the labeling process. The cells were incubated in a small test tube for about half an hour, without any mixing. Sedimentation can occur, and cells on top can prevent diffusion of mAbs to cells on the bottom. The blocking can lead to a large heterogeneity in label load, especially at the lowest concentrations of mAbs. However, there may be other causes, and it may be useful to understand the nature of this variability. The focus of this paper is the constant value of CV% at higher values of MFI, which suggests that the SD is proportional to MFI. The value of CV% will be related to a property of the cell, e.g., fluctuations in the number of bound labeled mAbs. The stochastic simulation was undertaken to understand the contribution of the labeling reaction to observed values of CV%, 22 ± 1 for FITC, and 17 ± 1 for APC, as shown in Figure 1a,b, respectively. In this manuscript, we are only considering cellular and molecular effects on the reaction dynamics and diffusion, but we are currently modeling effects of noise and gain in the measurement system, as well. The two stochastic models should allow the extraction of useful biological information from the CV% data.

### 2.2. Description of the Stochastic Model

**Figure 2:**
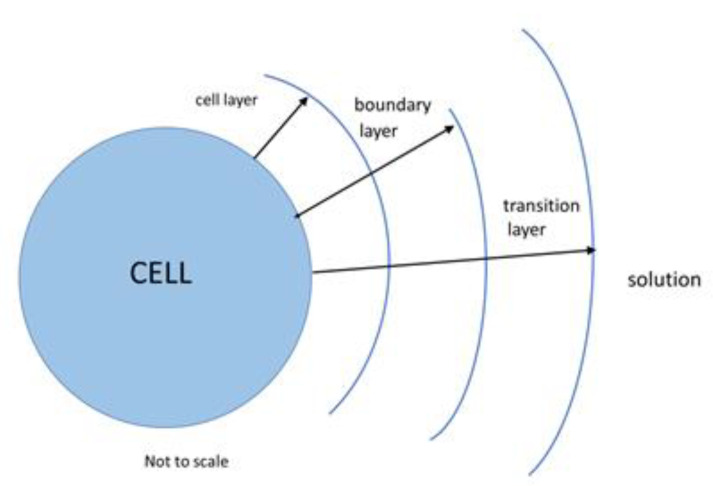
Geometry of the compartments used in the model. The surface of the cell is separated from the solution by three named layers. The “cell layer” contacts the surface of the cell and contains a layer of ions that neutralize the ions on the surface of the cell, the “boundary layer” defines the extent of the cell’s influence on solution bulk flow which is assumed to be zero, and the “transition layer” provides the initial condition for mass transport from bulk solution to the surface of the cell.

Application of stochastic reaction–diffusion models is an active research field with numerous recent developments [6]. There are several approaches and methods used to employ reaction–diffusion models and construct solutions to them [7]. While there are sometimes difficulties when employing such models because of irregularity of the noise terms that perturb the equation, such noise terms can quantify the lack of knowledge of certain parameters, finite-size effects, and/or fluctuations occurring due to external perturbations. In the present model, both the diffusion and the surface reactions are modeled as stochastic processes, using the Gillespie formalism [8,9], expanded to include diffusive mass transport [10]. During the labeling process, the mAbs in solution execute random walks and eventually encounter the surface of the cell where the mAbs are adsorbed and proceed to react with surface bound receptors. It is not practical to track the fluctuating spatial positions of thousands of mAbs, to estimate the times of their arrival at the surface of the cell. A practical method by which diffusion can be incorporated into the stochastic reaction algorithm was described in Reference [10]. In short, the space around the cell is partitioned into spherical thin shells, and the rate of transfer of one mAb between two contiguous spherical shells is given by D/h^2^, where D is the diffusion coefficient in unbound solution, and h is the thickness of the shell. In the current version of the model, all shells are 3.75 µm thick, about the average radius of the T cell. The solid blue circle in Figure 2 shows an idealized spherical cell with three surrounding spherical shells delineated by three solid curves. In the following, the spherical shells are referred to as layers.

In the current model, the bulk flow in the labeling solution is assumed to be zero so that the three layers simply provide a diffusive path for mAbs to reach the cell surface. However, the three layers serve as convenient placeholders for enhancements in the model. The layer bordering the cell, called the cell layer, *h_c_*, contains the counterions at the surface of the cell which shield the charged groups on the surface of the cell from the rest of the suspension. Once the mAbs enter the vicinity of the cell surface, the charged groups on the mAbs start to experience electrostatic interaction with the charged groups on the cell. Other int eractions may also occur as mAbs near the surface of the cell, resulting in nonspecific adsorption on the cell surface. The cell layer is the natural location for enhancements to the description of the absorption process. Adjacent to the cell layer, and further from the cell surface, lies the “boundary” layer in which any flow present in the bulk solution is influenced strongly by the presence of the cell. The boundary layer is a natural location to insert a phenomenological description of hydrodynamic mixing. The outermost layer is called the “transition” layer; it defines the initial conditions for mass transport from bulk suspension to the surface of the cell. In addition, the transition layer is a placeholder for extensions of the model to lower concentrations of labeling mAbs. In the current model, the number of mAbs in the transition layer is set to a constant value derived from the large concentration of mAbs in the bulk suspension. The large concentration is needed to ensure saturation of binding and ensure that the concentration of mAbs in the suspension does not change appreciably during the labeling process. In case of a smaller initial mAb concentration in the labeling suspension, a feedback loop is needed to adjust, downward, the number of mAbs in the transition layer, to reflect the decrease in suspension concentration due to the binding of mAbs to receptors on the surface of T cells. In the present model, the mass transfer between the three layers is diffusive, resulting in the smallest possible mass transfer to the surface of the cell.

The mAb binding process starts with the transport of mAbs to the surface of the cell. The relevant dynamic variables are the number of mAbs in each of the layers and on the surface of the cell. The number of mAbs in the transition layer is represented by the symbol (A_s_), where the subscript “s” is a reminder that the transition layer properties are those of the labeling suspension. The number of mAbs in the boundary layer is represented by (A_b_), and the symbol (A_c_) stands for the number of mAbs in the layer adjacent to the cell surface. The dynamic variable called (A_o_) represents the number of mAbs adsorbed on the cell surface via nonspecific interactions. The specific reaction between the adsorbed mAbs and CD4 surface receptors, denoted by (R), is described by the dynamic variables (AR) and (ARR) which represent the number of mAb bound to one and two CD4 receptors, respectively. The symbol (R) represents the number of unbound CD4 receptors on the cell surface. It was assumed that, at the start of the binding reaction, there were 100,000 unbound receptors on the cell [5]. The model’s conceptual framework is represented below by the five transitions/reactions which describe the motion of mAbs from solution to the cell surface and then to the complexes (AR) and (ARR). In all, there are ten sequential reactions, i.e., five forward and five backward, as shown in the list below.
(*A_s_*) ⇆ (*A_b_*)diffusion between transition and boundary layers(*A_b_*) ⇆ (*A_c_*)diffusion between boundary and cell layers(*A_c_*) ⇆ (*A_o_*)nonspecific adsorption—cell layer to surface(*A_o_*) (*R*) ⇆ (*AR*)monovalent binding of mAb and receptor on surface(A*R*) (*R*) ⇆ (*ARR*)bivalent binding of (R) and (AR) on surface


The first column in Table 1 gives the values of the reaction parameters associated with the ten reactions. The second column describes the reaction associated with the reaction parameter. The symbol D represents the mAb diffusion coefficient, which is approximately 0.5 × 10^−10^ [11]. The symbols h_c_, h_b_, and h_t_ give the thickness of the cell layer, boundary layer, and transition layer, respectively. The reaction rate constants, starting with Row 5 in Table 1, are given in units of 1/s and can be interpreted as the probability that a single molecule will undergo the event associated with the reaction in an infinitesimal time interval. The reaction rate constants describing diffusion are obtained by dividing the solution diffusion coefficient by the square of the dimension of the layer from which the molecule is diffusing.

It is important to point out that the adsorption process in Table 1 is simplified. The reaction constant k_on_ in Table 1, is simply the diffusion rate, meaning that every molecule that comes to the surface is adsorbed by the surface and there is no reflection. The rate constant k_off_ was set to 0.5 × k_on_, to ensure that the number of unoccupied adsorption sites remained reasonably constant during the labeling process and that there was always an ample number of adsorbed mAbs. (The current model assumes that the number of nonspecific adsorption sites on the surface of the T cell is very large, so that it does not have to be explicitly considered as a dynamic variable.) The values of the reaction constants, k_mp_, k_mn_, k_bp_, and k_bn_, were chosen to yield a reasonable time variation of the variables (R), (AR), and (ARR) in the labeling process. The values of the four reaction constants were changed in the calculations below, to describe special cases dominated by monovalent or bivalent binding. The value of (A_0_) controls the reaction rate of monovalent binding, so a larger (A_0_) results in more (AR), which in turn depletes the number of adsorbed mAbs and allows more mAbs to enter the adsorption state from the cell layer. At equilibrium, the number of adsorbed mAbs is equal to about 6000 = (k_on_/k_off_) × (A_c_) = 2 × (A_c_). At equilibrium, the cell layer, the boundary layer, and the transition layer all contain about 3000 mAbs, on the average (calculated by multiplying the volume of the transition layer (4.76 × 10^15^ m^3^) by the concentration of mAb in the bulk suspension (1 µmol/m^3^) and Avogadro number; the result is rounded to 3000).

For reactions between two molecules, the reaction rate constant is the probability that a pair of molecules will undergo the event associated with the reaction in an infinitesimal time interval. In practice, there are many pairs of molecules in each of the states described in Figure 2. The total reaction rate (called the propensity function) can be found by multiplying the reaction rate constant given in Table 1 by the number of pairs undergoing the reaction. The result is shown in Table 2, where the symbol α stands for propensity function, as defined in Table 2, and the subscripts identify the specific reaction, which is described in the rightmost column of Table 2.

The stochastic nature of the model is evident in the method by which the time of occurrence of any one of the ten reactions is chosen, and the method is given by the Gillespie algorithm [8]. In short, for any one of the reactions given in Table 2, the probability that the reaction will take place at time *t* + *τ* + *dτ*, if it did not occur by time *t* + *τ*, is given by the following:

exp(−*kA*(*t*)*τ*) = exp(−*α*(*t*) ∙ *τ*)

where *A*(*t*) is the number of molecules at time (*t*), and *dτ* is an infinitesimal time interval. Let *r*_1_ be a random number chosen uniformly between 0 and 1, and then the time at which the reaction occurs is given by *τ* = (1⁄*α*(*t*)) ∙ ln (1⁄*r*_1_), where *τ* is distributed uniformly between 0 and ∞. The probability that any one of the ten reactions in Table 2 occurs at *t* + *τ* + *dτ* can be written as exp (−(∑ *α_i_* (*t*)) ∙ *τ*), where it is assumed that the numbers of all molecules are known at time, *t*. In that case, the time of occurrence of any one of the ten reactions is given by Equation (1).

*τ* = (1⁄∑ *α_i_* (*t*)) ∙ ln (1⁄*r*_1_). (1)

The occurrence of a specific reaction is found by examining the fractions *α_j_*(*t*)⁄∑ *α_i_* (*t*). The sum of all the fractions is equal to 1. It is useful to visualize the probability of the occurrence of a reaction at *t + τ* by a line segment of length 1; the likelihood of any specific reaction is given by the line segment of length equal to the fraction associated with that reaction. Therefore, the reaction can be selected by choosing a random number distributed uniformly from 0 to 1 and determining into which line segment it falls. When the reaction occurs, all relevant variables change value by one. The “change” matrix is defined in Table 3. The first column is the variable vector containing the numbers of the six named variables, and the first horizontal row gives the reaction name denoted by the reaction rate constant. Each column of the change matrix is a vector which is added to the variable vector to give an updated variable vector after the reaction takes place. For example, if reaction k_mp_ occurs, then variables (A_o_) and (R) decrease by one, and variable (AR) increases by one.

The diffusion in the stochastic model involves the transfer of one mAb between two adjacent layers; for example, for reaction k_1p_, one mAb goes from boundary layer to cell layer. There are many mAbs in each layer, and each mAb is constantly executing a random walk, and, on occasion, an mAb will stray into a neighboring layer. That event is called a “reaction”. Note that the “reactions” k_2p_ and k_2n_ describe diffusion between the transition and boundary layers, and the population of transition layer is set to 3000. The stochastic model is implemented as follows. At a given time, all the propensity functions, *α*, shown in Table 2, are calculated. A random number, *r*_1_, is used to calculate the time of occurrence of the next reaction by Equation (1). The reaction that occurred is determined by choosing a random number, *r*_2_, and finding where, as determined by *α_j_*(*t*)⁄∑ *α_i_* (*t*), it falls in (0,1). Finally, the column of the change matrix associated with the reaction is added to the variable vector. Lastly, time is incremented by the value *τ* given by Equation (1). Upon completion, the entire cycle is repeated. The following section describes the results of applying the model to the reaction between labeled mAbs and CD4 receptors on the surface of T cells.

### 2.3. Characteristics of the Results from the Stochastic Model Calculation

**Figure 3:**
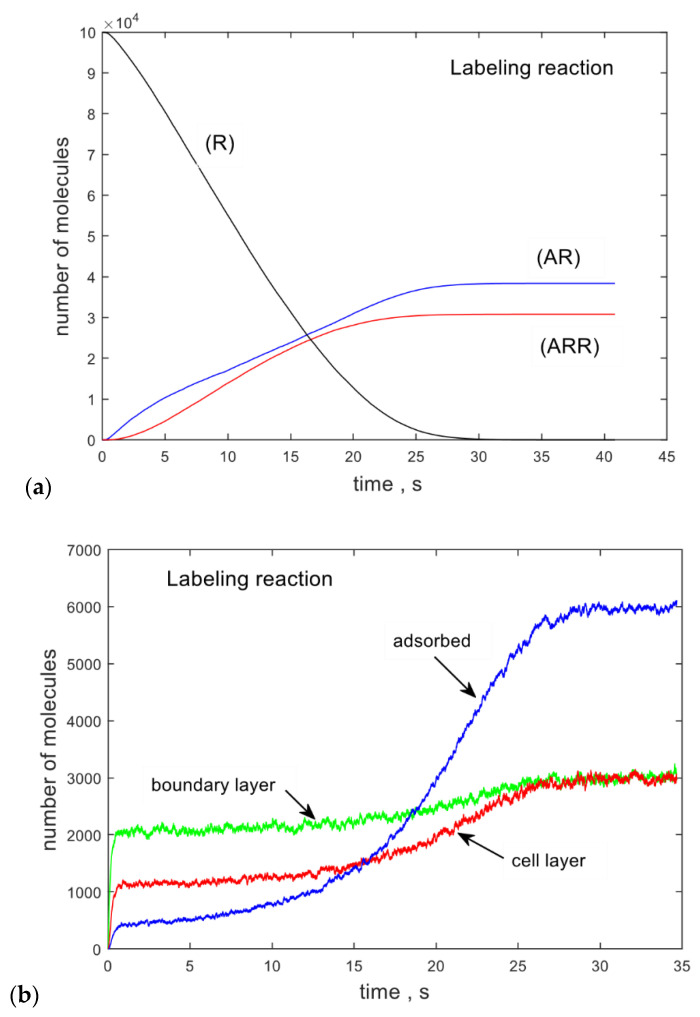
(**a**) The labeled traces show the time dependence of the surface-bound populations in the labeling reaction. These include (R), the surface unbound receptors, and (AR) and (ARR), the monovalent and bivalent complexes of R and A. (**b**) The labeled traces show the time dependence of the mAb populations of layers surrounding the cell and providing a path for the transport of mAbs to the surface of the cell. The layers are defined in Figure 2.

Figure 3a,b shows results of stochastic model calculation for initial conditions (R) = 100,000, (A_t_) = 3000, and all other variables set to 0. The reaction constants, k_mp_ = 10^−4^, k_mn_ = 10^−6^, k_bp_ = 2 × 10^−6^, and k_bn_ = 10^−8^, were chosen to yield comparable values of (AR) and (ARR) at equilibrium. All the other reaction parameters were those given in Table 1. Figure 3a shows the time dependence of mAb populations on the cell surface. The trace labeled (R) gives the total number of unreacted CD4 receptors on the T cell. As expected, it decreases as the labeling reaction proceeds. The labeling reaction on the surface leads to (AR) (monovalently bound mAb) and (ARR) (bivalently bound mAb). The time dependence of these populations is shown by the blue and red curves labeled (AR) and (ARR). At some point, the receptors are consumed and (R) = 0. At this point, the curves (AR) and (ARR) flatten.

Figure 3b shows the time dependence of populations involved in mass transport of mAbs to the cell surface. The trace labeled “boundary layer” shows the number of mAb molecules in the boundary layer surrounding the cell. The boundary layer exchanges mAb molecules with the transition layer and the cell layer via diffusion. The transition layer provides the initial condition for mass transport to the cell surface, and, in this version of the model, the number of mAbs in the transition layer is constant.

**Figure 4:**
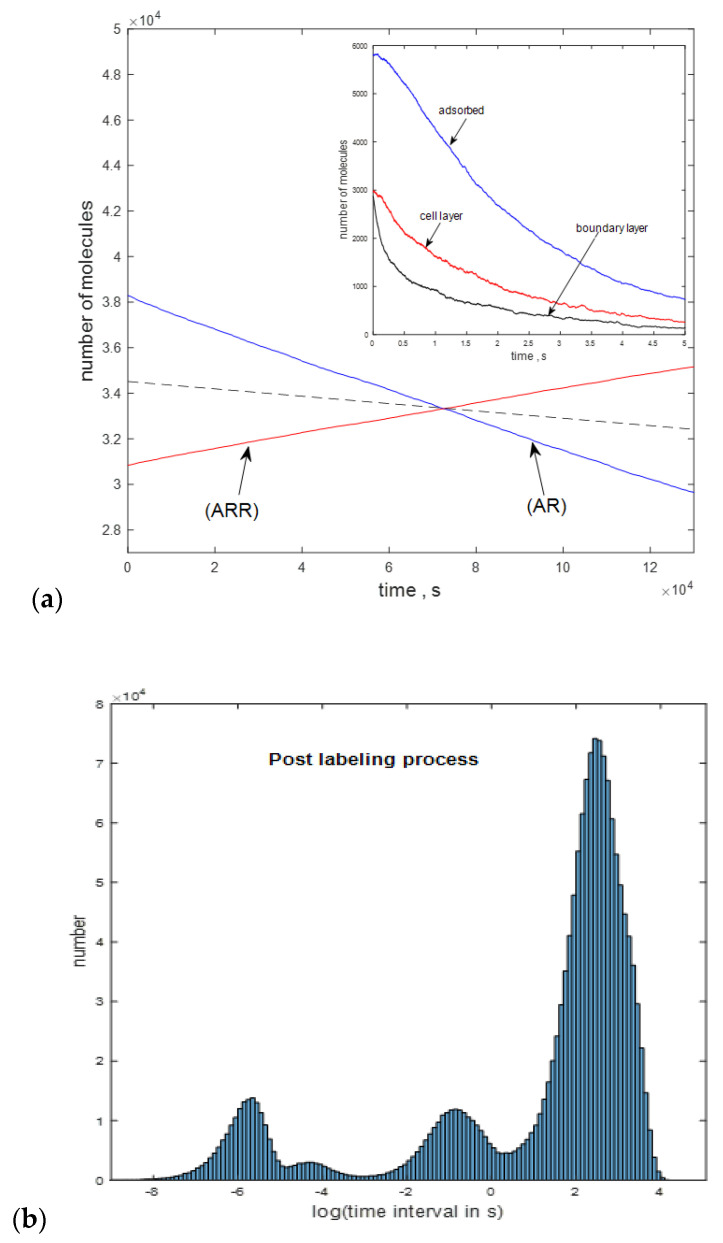
(**a**) The evolution of the labeling reaction populations after the labeling reaction was stopped. The dashed line is proportional to the sum (AR) + (ARR) = (ABC); (**b**) distribution of time intervals between reactions in the post-labeling process. The long intervals are associated with the dissociation reactions of AR and ARR. ABC = antibodies bound to the cell.

After a rapid growth during the first 1 s, the number of mAbs in the boundary layer undergoes a very slow growth because a slightly larger number of mAbs diffuse into the boundary layer from the transition layer than diffuse out of the boundary layer and into the cell layer. The label (A_c_), shown by the red trace, gives the number of mAbs in the cell layer. Finally, the number of mAbs adsorbed on the cell surface is given by the blue trace labeled ”adsorbed”. The numbers of mAbs in the boundary layer and the cell layer reach a new equilibrium value at about 25 s. This time marks the transition to labeling equilibrium, where the total number of bound receptors is given by (AR) + 2∗(ARR), which should equal the initial number of unbound receptors (R) set to 100,000 [12]. Though 140,000 CD4 receptors on cryopreserved peripheral blood mononuclear cells (PBMCs) were quantified by using quantitative mass spectrometry [5], not all of these receptors are accessible for affinity binding by mAbs, as demonstrated by flow cytometry measurements. The stochastic model calculation produces a large file containing times at which the individual reactions occurred. The time intervals between consecutive reactions can be used to make a histogram. The time intervals between reactions range between 0.1 ns and 10 ms, and there is an indication of two clusters in the histogram of time intervals. The clusters may be assigned to specific reaction groups, but, at present, there is no practical way to measure the time intervals between reactions. Of great practical interest is the course of the post-labeling reaction after the labeled mAbs have been separated from the labeling solution and resuspended in a buffer without any mAb. Figure 4a shows the model calculation with the initial values of the variables set to the final values of the variables from the calculation shown in Figure 3a. The reaction parameters were the same as in Figure 3a.

Note the large difference in the time scale of the horizontal axis in Figure 4a, as compared to the time scale in Figure 3a. The forward reaction, shown in Figure 3a, reached equilibrium in 25 s, while the backward reaction, shown in Figure 4a, goes to 3 × 10^4^ s, without reaching an equilibrium. Of greatest interest is that (AR) and (ARR) populations exchange mAbs very readily between themselves, resulting in a quasi-constant value of the total bound mAb, (ABC) = (AR) + (ARR). The calculation indicates that, even after 2 h (7200 s), the number of bound mAb, remains close to the initial value at the end of the labeling process. This means that the washing and resuspension of labeled cells may not have a significant impact on the measured MFI, which is determined by the value of (ABC). The large time scale of the decrease in the (AR) and (ARR) populations, and the nearly linear behavior of the decrease, may permit measurement of the two populations over time, determination of the slopes of the two curves, and extrapolation to initial time to obtain the numbers of the two populations at the end of the labeling process. These populations would permit the conversion of ABC to a quantity proportional to bound receptors given by (AR) + 2 × (ARR). However, the result of the model calculation may not be generally valid for all mAb binding reactions. Figure 4b shows the histogram of time interval data for the calculation in Figure 4a. A histogram formed by using data at the start of the calculation was dominated by time intervals between 10^−6^ and 10^−4^ s, whereas histograms formed by using time intervals from the end of the calculation were dominated by time intervals around 2 s. At the beginning of the calculation, the main reactions were those of mAbs desorbing from the surface and diffusing to the boundary layer and subsequently to the solution. Time intervals at the end of the calculation were dominated by the slow backward reactions involving (R), (AR), and (ARR). The histogram in Figure 4b suggests that the algorithm is effective at selecting the sequence of reactions during the post-labeling process (and hence also during the labeling process). Calculations were also performed with rate constants tailored to maximize the production of (AR) or the production of (ARR). In both cases, the predicted reduction in bound mAb after washing and resuspension was minimal.

### 2.4. Results of Calculations with Different Sets of Model Parameters

The stochastic model, described in Section 2.2 and Section 2.3, was used to estimate the CV% of (ABC) for various reaction conditions. For each set of reaction conditions, the calculation was repeated 25 times, and the CV% was obtained from the mean and the standard deviation of the 25 (ABC) values. The number 25 was a compromise between achieving a reasonable estimate of CV% and keeping the calculations to a manageable period of time. Section 2.4.1. gives the results of calculations with a constant set of reaction parameters. This CV% reflects the fluctuations associated with the random selection of reaction sequence. In Section 2.4.2., the number of CD4 receptors varies for each of the 25 calculations, appropriate for a cell population with varying number of receptors on each cell. Section 2.4.3 gives the results of calculations with varying values of bivalent reaction constant, and finally with varying values of both the bivalent reaction constant and the number of receptors on the cell. As expected, the CV% of (ABC) increases as the number of fluctuating reaction properties increases. Section 3 discusses the selection of the most relevant set of fluctuating properties to include in the stochastic model describing mAb binding to CD4 receptors on the surface of T cells.

#### 2.4.1. CV% for Reactions under Constant Conditions

The first estimate of CV% for (ABC) was performed with constant reaction conditions. A set of 25 calculations was performed, fixing the number of receptors on the cell surface at 100,000, and the reaction constants given in Table 4. This was appropriate for a comparable mix of monovalent and bivalent binding. The results of 25 calculations are summarized in Table 4.

**Figure 5:**
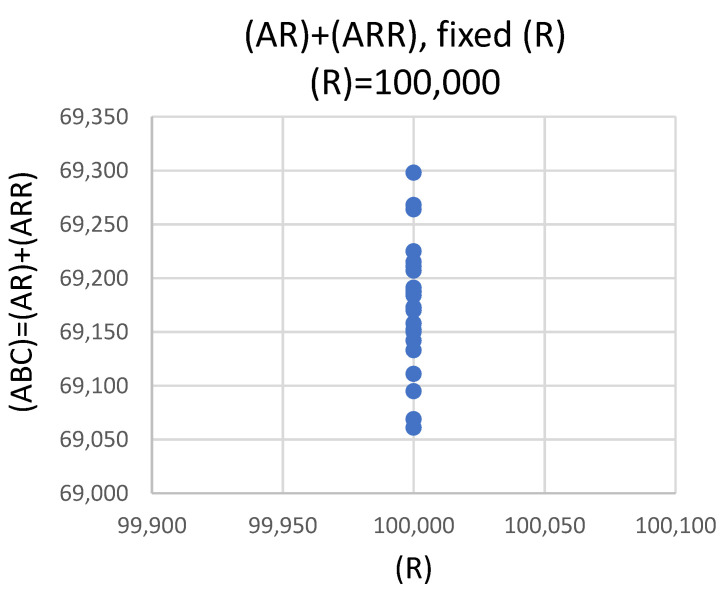
Equilibrium values of (AR) + (ARR) at the end of 25 calculations of the stochastic model. The values (AR) and (ARR) are correlated to yield a very small CV% for (ARR) + (AR). The CV% for (AR) and CV% for (ARR), taken individually, are much larger.

The value of CV% for (AR) + (ARR) = Total(A) is negligible, in contrast to much larger values of CV% for (AR) and (ARR) separately. The reason for this curious behavior is that, at equilibrium, (AR) and (ARR) are correlated through the constraint 100,000 = (AR) + 2∗(ARR). The steps in the stochastic model ensure this underlying constraint is satisfied because the reaction rates to produce (ARR) and (AR) depend on (R); thus, the reaction, which uses up the last free receptor, stops the other reaction from continuing. At saturation ((R) = 0), (AR) and (ARR) are inversely proportional; that is, if (AR) increases, then (ARR) must decrease, and vice versa. The conclusion is that the intrinsic reaction dynamics contribute little to the CV% of (ABC) = (AR) + (ARR) and can be neglected. Figure 5 demonstrates this behavior.

The value of (AR) + (ARR) in Figure 5 ranges from 69,050 to 69,300, with a CV% of about 0.1%. Note that the scale of the *y*-axis in Figure 5 is very fine, i.e., 50 units per gradation.

Both monovalent and bivalent binding yield one bound mAb on the surface of the cell. However, bivalent binding consumes two receptors, while monovalent binding uses a single receptor. Consequently, for a fixed number of receptors on the surface of the cell, bivalent binding yields (ABC) value, which is half of the value obtained for monovalent binding. If both types of bindings are present in comparable amount, the resulting (ABC) is between the (ABC) values obtained for monovalent and bivalent binding. Therefore, it is important to determine the mix of monovalent and bivalent binding in a population of cells. Recent modeling of the labeling of FITC-mAb and APC-mAb to CD4 receptors on T cells suggested that, while FITC-mAb binding was mostly monovalent, that of APC-mAb was mostly bivalent [13]. In this case, it was observed that the concentration of labels needed to reach saturation was much higher in the case of FITC-mAb than in APC-mAb. If the cells in the population have a different mix of monovalent and bivalent binding, the (ABC) values for that population would exhibit an increased CV%. This would be a significant contribution to CV% caused by intrinsic reaction dynamics. It is investigated in Section 2.4.3., but first we look at the CV% of (ABC) for a population of cells with a variable number of receptors (but fixed values of all reaction constants).

#### 2.4.2. CV% due to Fluctuating Number of Receptors on the Surface of the Cells

The CV% of the number of bound mAbs for a cell population may vary due to fluctuation in the number of accessible CD4 receptors on the T-cell surface. Calculations were performed for a series of cells with CD4 receptor numbers chosen from a normal distribution with a mean of 100,000 and a CV% of 6%. The results of the calculations are summarized in Table 5. The mean and standard deviation (SD) for the three cases were obtained from 25 calculations each.

**Figure 6:**
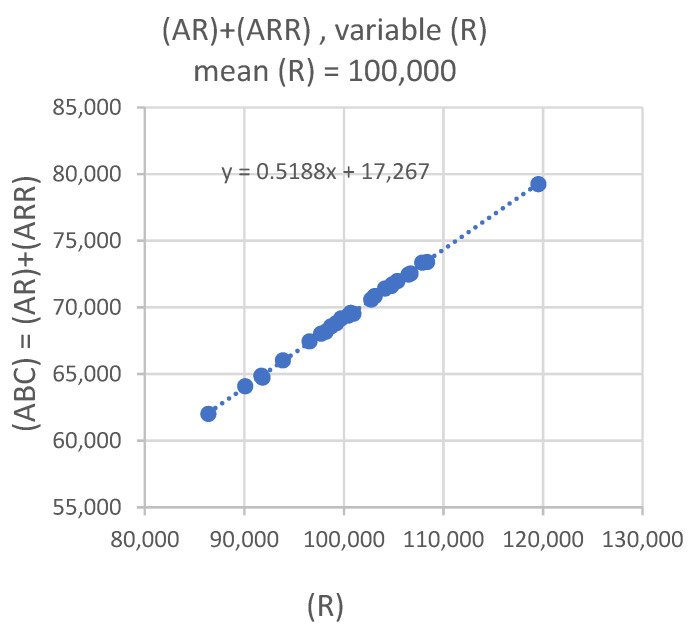
(AR) + (ARR) equilibrium values for the same calculation as in Figure 5 with the exception that the initial value of ^®^ for each calculation is taken from a normal distribution, with a mean of 100,000 and a CV% of 6%. Note the scale of the y-axis in Figure 6 is much larger (5000 units per gradation) than the scale of y-axis in Figure 5 (50 units per gradation).

The solid circles in Figure 6 show the results of 25 model calculations for the case where (AR) and (ARR) have similar populations at equilibrium but the cells have varying number of receptors (first entry in Table 5). Each solid circle represents the result of a single calculation, and the diameter of the solid circle gives the intrinsic fluctuation for (ABC) = (AR) + (ARR) due to the stochastic process (shown in Table 4). The values of (ABC) in Figure 6 depend linearly on the values of (R), shown on the horizontal axis, and the points cluster around the mean value of 70,000, which is equal to the sum of the means of (AR) and (ARR). The distribution of (ABC) on the vertical axis in Figure 6 follows the distribution of (R) on the horizontal axis. There is an appearance of the *y*-scale of the plot in Figure 5 expanding and the plot being tilted to the right to yield Figure 6. Clearly, the fluctuations in the number of receptors on the surface of T cells determine the fluctuation of (ABC) and hence the CV% of (ABC). Table 5 also shows the CV% of (AR) + (ARR) for the case where monovalent or bivalent binding dominate separately. In both cases, the CV% is slightly larger than that shown in Figure 6. The average CV% for the three cases shown in Table 5 is about 6%.

#### 2.4.3. CV% in the Case of Fluctuating Bivalent Binding Affinity and the Number of CD4 Receptors

Reactions between molecules inside cells [14] and on cell surfaces [15] are affected by molecular crowding caused by the molecular milieu. Molecular crowding could occur if surface receptors cluster to modulate their response to some signaling event. The mAbs arriving at a cluster of receptors may undergo monovalent binding to one of the receptors in the cluster. The monovalent binding in turn facilitates bivalent binding because of the large number of receptors in the vicinity [16]. It is expected that some of the CD4 receptors on T cells may form clusters, and the extent of clustering will be different on different T cells. In order to estimate the effect of clustering on measured CV%, it was assumed that there are two different types of CD4 receptors: those in clusters and those that are solitary, and each T cell is populated by either solitary or clustered CD4 receptors. This scenario is extreme, and it only serves to estimate an upper limit for the effect of clustering. In addition, it is assumed that the bivalent binding to CD4 in clusters is stronger than the bivalent binding for solitary receptors. In the calculation, k_bp_ is set to 10^−6^, to describe the bivalent binding in clusters, and set to 10^−7^, to describe the binding of mAbs to solitary CD4 receptors. The value of k_bn_ remains at 10^−9^ for both cases. Such a change in reaction constant is plausible since it represents a decrease in free energy of only 1.4 kJ/mol [17]. Table 6 shows the results of 25 calculations, where the value of k_bp_ for a given T cell was chosen randomly to be either 10^−7^ or 10^−6^. The number of receptors was held at 100,000, and the reaction constants were chosen to populate both AR and ARR states.

**Figure 7:**
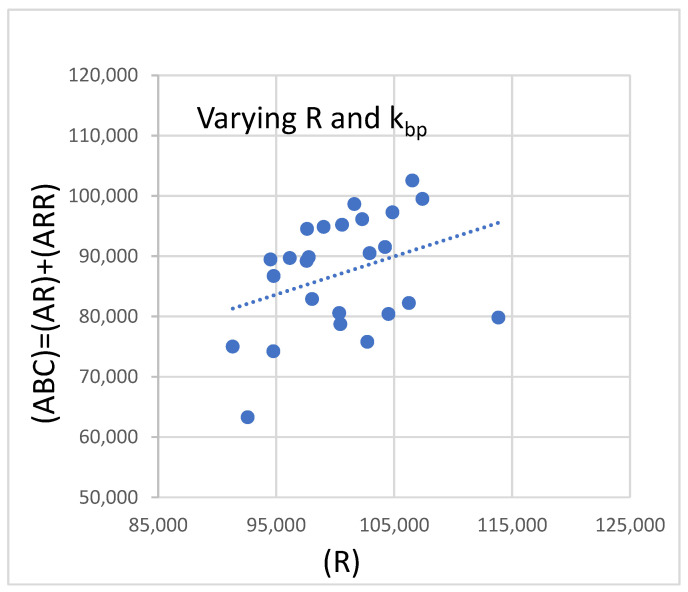
Equilibrium values of (AR) + (ARR) for the same calculation as in Figure 6, with the exception that the value of k_bp_ for each calculation is taken from a normal distribution with a mean of −6.5 and a CV%. of 8. In addition, the initial value of (*R*) fluctuates, as shown in Table 6.

The CV% of the total bound mAb population is around 11.1%. The value of (AR) varies between 65,000 and 113,000, while the value of (ARR) jumps between 24,000 and 7400. The CV% for the individual populations is much larger, 26.5% for (AR) and 69% for (ARR); however, the CV% of the combined population is only 11%. This occurs because the constant value of (R) for all T cells correlates with the production of the AR and ARR states. The relationship between (AR) and (ARR) is inversely proportional. The CV% of 11% was obtained for the extreme case where each T cell had a bivalent binding constant, k_bp_, equal to either 10^−6^ or 10^−7^. The above scenario may be unlikely; however, the surface properties of the T cell are complex, and it would be expected that the average reaction constants vary from cell to cell. The more likely scenario, where the exponent of k_bp_ is distributed normally with a mean of −6.5, and an SD of 0.5 gives the results shown in Table 7. The CV% of (ABC) = (AR) + (ARR) is 11%. Figure 7 shows the plot of (ABC) versus (R) for the calculations in Table 7.

Unlike the plot in Figure 6, the values of (ABC) in Figure 7 have a more random distribution, although there is some clustering of points above and below the dashed trend line shown in Figure 7. The surfaces of T cells are populated by a varied selection of biomolecules, and it is expected that, in addition to k_bp_, other reaction constants will also have varying values for each T cell. It is easy to devise combinations of varying reaction parameters which give 15% or more for the value of CV% of (ABC). The chosen parameter variations must be based on known or expected physical properties of the T-cell surface.

Fluctuations in cell radius, and hence cell surface area, may also contribute to the measured CV% of MFI. The diameter of the cells in the PBMC sample was measured at 7.5 ± 0.4 µm [5]. The implied variation in cell area is 20% and can influence the number of adsorbed mAbs, since the total number of adsorbed mAbs is expected to be proportionate to the area of the cell. The effect of changing number of adsorbed mAbs at equilibrium can be estimated by changing the parameter k_off_ in the model. Larger values of k_off_ signify a greater desorption rate, resulting in less adsorption at equilibrium, and smaller values of k_off_ will result in greater adsorption. Changes in value of k_off_ from 0.4 to 0.6 result in changes of 1.5% in (ABC) at equilibrium, where equilibrium is defined as the end of the calculation. Increasing the length of the calculation from 30 to 40 s reduces the changes in (ABC); hence, the fluctuations in (ABC) depend on closeness to equilibrium. This suggests that changing the number of adsorbed mAbs changes the time needed to reach equilibrium but does not appreciably change the value of (ABC) at equilibrium. The time of labeling is 30 min, suggesting that equilibrium will have been reached for all cells with different radii or different amounts of adsorbed mAb. Thus, fluctuations in the cell radius are not expected to contribute to fluctuations in (ABC) values.

## 3. Discussion

The stochastic binding model was created to support the development of standards for flow cytometer measurements. CD4^+^ T cells fulfilled the role of a standard because the number of CD4 receptors on CD4^+^ T cells is reasonably constant for all healthy individuals. In addition, due to their central role in adaptive immune response, CD4^+^ T cells have been extensively studied, and CD4^+^ T-cell preparations were available commercially. Modern flow cytometers can measure more than 12 different mAb labels specific to more than 12 different receptors on the surface of the cell. The fluorescence response from each labeled mAb is measured in one of the different fluorescence channels of the flow cytometer. To standardize the fluorescence response of the different fluorescence channels, the antibody specific to CD4 must be labeled with different labels appropriate for the fluorescence channels. Clearly it is important to have a basis for comparing the binding of CD4 receptors to mAbs labeled with different dyes. A previous work analyzed the dependence of MFI on the initial concentration of labeled mAbs in the labeling solution [13] and found that the labeling of mAbs with APC was dominated by bivalent binding, while the labeling of mAbs with FITC was dominated by monovalent binding. The stochastic-binding-model calculation indicated that differences in the amount of monovalent and bivalent binding of the mAbs to the CD4^+^ T cell lead to significant differences in the detected MFI. The binding is a complex process which is under intense study due to its central role in human adaptive immune response, which leans heavily on the ability of T-cell surface receptors to recognize antigens [17]. Three stages in the binding process have been identified. The first stage starts with the arrival of the labeled mAbs on the surface of the cell and the start of the sampling of the free energy landscape. At some point, the mAb arrives at the proximity of the T-cell receptor (to which it is matched) and experiences a larger negative free energy change. This makes it more likely for the mAb to remain in the vicinity and continue sampling the local conformations. At some point, the free energy change is sufficiently negative and stable so that the mAb and the receptor can enter an “induced fit”, during which their conformations change in a coordinated way, to reach an optimal negative free energy. The “induced fit” process is intimately coupled to solvation water dynamics [18]. Most likely solvation water dynamics is also involved in the process of “sampling the free energy landscape” [19,20]. A slightly modified sequence of events describes bivalent binding. To explore the stability of the ratio between monovalent and bivalent binding, we analyzed the dependence of CV% on MFI at binding saturation. The CV% was 22 ± 1 for FITC and 17 ± 1 for APC. After subtracting an assumed contribution of 5.2 due to fluorescence detection (obtained from a stochastic model of fluorescence response of a flow cytometer with a flat-top laser), the values of binding CV% become 21.5 ± 1 for FITC and 16.1 ± 1 for APC. These values represent the upper bound on the sum of the CV% for the binding steps described above. The model suggests that the main contribution to the binding CV% is the fluctuation of the number of receptors on the PBMC, with lesser contributions from fluctuations in the binding properties. The large difference in CV% between FITC, a small molecule, and APC, a large fluorescent protein, suggests that the size of the label might play a role in the binding process. The difference may help to separate the contributions to CV% due to fluctuations in the number of mAbs and fluctuations due to their binding properties. However, this hypothesis needs to be verified by more measurements with dye molecules with different sizes. The development of CD4^+^ T-cell-based reference material for calibrating flow cytometer measurements has facilitated the conversion of the MFI scale to the ABC scale [4]. Thus, in addition to the average value of ABC, the flow cytometer measurement can also provide the associated SD. The interpretation of the binding CV% in terms of fluctuations of bound mAb provides an additional tool for the study of fluctuations of functionality of CAR-T cells similar to the fluctuations observed in CD8+ T cells [21]. This will help to understand the observed variability of the response to CAR-T-cell therapy. The binding CV%, along with MFI, will play a role in the applications of flow cytometry to quality control in the manufacture of CAR-T cells [22].

## 4. Materials and Methods

The measurements used in this manuscript were thoroughly described in a previous work [13]. In short, a 100 µL aliquot of thawed peripheral blood mononuclear cells (PBMCs; Cellular Technology, Shaker Heights, OH, USA), at approximately 10^7^ cells/mL, was added to a defined volume of labeled CD4-FITC, SK3 clone (BioLegend cat. no. 344604) or CD4-APC, SK3 clone (BioLegend cat. No. 344614) mAb solution in a test tube prewetted with Phosphate Buffer Solution (PBS), and 2% Fetal Bovine Serum (FBS; Sigma-Aldrich, St. Louis, MO, USA). To obtain the MFI curve (Figure 1), we used the manufacturer’s recommended amount as the initial concentration of mAbs, followed by eight 2-fold dilutions and one 1.5-fold dilution. The suspension of labeled mAb and cells was vortexed to mix for a few seconds and then incubated for 30 min, in the dark, at room temperature (22 °C), without additional mixing. After the incubation time, the cells were washed with 2 mL of the PBS and 2% FBS buffer, centrifuged, and re-suspended in 0.5 mL of the same buffer. The stained PBMCs were analyzed by using an Aria II flow cytometer within 2 h after the preparation of the sample. The fluorescein isothiocyanate (FITC) and allophycocyanin (APC) labels were measured, using the FITC and APC fluorescence channels of the Aria II flow cytometer (BD Biosciences, San Jose, CA, USA). Data were analyzed by using BD FACS Diva (BD Biosciences, San Jose, CA, USA). The gating of CD4^+^ lymphocytes was also confirmed with forward and side scatter and by staining of both CD3 V450 and CD4 FITC or APC in some cases.

## 5. Conclusions

A stochastic reaction–diffusion model was developed to describe the binding of labeled monoclonal antibodies (mAbs) to CD4 receptors on the surface of T cells. The labeled mAb and the T cells were mixed in a buffer and left to interact for about 30 min. During this period, the mAb diffused to the surface of the cell, adsorbed on the surface, and proceeded to bind the CD4 receptors. The diffusion was modeled by introducing three layers between the bulk suspension and the surface of the cell. The cell surface exhibited perfect absorption, meaning that all mAbs reaching the cell surface were adsorbed. The desorption rate was set of 0.5 of the absorption rate, to give a reasonable number of adsorbed mAbs and, at the same time, ensure that the adsorption sites on the surface of the cell were not significantly depleted, in accordance with the model assumption. The adsorbed mAb interacted with the CD4 receptors forming monovalent, (AR), and bivalent, (ARR), complexes. The MFI measured by the flow cytometer was proportional to the total number of mAbs, given by (AR) + (ARR) = (ABC). The fluctuations in the number of bound mAb on the surface of the cell contributed directly to the CV% of the measured MFI. The analysis presented above suggests that the fluctuations in the number of mAb receptors on the T cell contribute about 6% to the CV% of MFI (Table 5). Including fluctuations in the bivalent binding constant, k_bp_, we get a larger CV% of 11% (Table 6). Given the complexity of the T-cell surface, it is expected that other reaction constants, e.g., k_mp_, will also exhibit fluctuating values on different T cells and contribute to the CV% of (ABC) and MFI.

Experience gained in the initial modeling effort suggests that the observed CV% of 17% to 22%, shown in Figure 1, can result from fluctuations in the values of reaction constants, fluctuations in the number of available CD4 receptors on T cells, and fluctuations introduced by fluorescence detection process. Since the fluorescence detection and the labeling reactions are independent processes, the square of the measured CV% is equal approximately to the sum of the squares of the CV% contributions from the fluorescence detection and labeling reaction processes. It is important to identify the CV% contribution from the fluorescence detection process alone and identify the contribution from fluctuations associated with the labeling reaction. MFI is proportionate to the laser illumination, average absorption cross-section, and the average quantum yield of the labels on the cell. Fluctuations in the value of these factors contribute to the CV% of MFI. Preliminary calculations with a stochastic model of the fluorescence detection process gave a likely contribution of 7% (for a flow cytometer equipped with a laser with a gaussian beam) to the total CV% of MFI, implying a 19% contribution from fluctuations in T-cell reaction properties. The large contribution provides an impetus to identify and quantify the biological fluctuations.

## Figures and Tables

**Table 1:** Values of parameters and reaction rate constants used in the model.

h_c_ = 3.75 × 0^−6^	layer adjacent to cell surface, m
h_b_ = 3.75 × 10^−6^	boundary layer, m
h_t_ = 3.75 × 10^−6^	transition layer, m
D = 0.5 × 10^−10^	diffusion coefficient, m^2^/s
k_2p_ = D/h_t_^2^	from transition to boundary layer, 1/s
k_2n_ = D/h_b_^2^	from boundary to transition layer, 1/s
k_1p_ = D/h_c_^2^	from boundary layer to cell layer, 1/s
k_1n_ = D/h_d_^2^	from cell layer to boundary layer, 1/s
k_on_ = D/h_c_^2^	from cell layer to surface adsorption, 1/s
k_off_ = (0.5) × k_on_	desorb from surface to cell layer, 1/s
k_mp_ = 1.0 × 10^−4^	from A and R to AR complex, 1/s
k_mn_ = 1.0 × 10^−6^	from AR complex to A and R, 1/s
k_bp_ = 2.0 × 10^−6^	from AR and R to ARR complex, 1/s
k_bn_ = 1.0 × 10^−8^	from ARR complex to AR and R, 1/s

**Table 2:** Values of ten reaction propensity functions used in the model.

α_2p_ = k_2p_ × (A_s_)	from transition to boundary layer
α_2n_ = k_2n_ × (A_b_)	from boundary to transition layer
α_1p_ = k_1p_ × (A_b_)	from boundary layer to cell layer
α_1n_ = k_1n_ × (A_c_)	from cell layer to boundary layer
α_on_ = k_on_ × (Ac)	from cell layer to surface adsorption
α_off_ = (0.5) × k_on_ × (A_o_)	desorbs from surface to cell layer
α_mp_ = k_mp_ × (A_o_)(R)	production of AR complex
α_mn_ = k_mn_ × (AR)	degradation of AR complex to A and R
α_bp_ = k_bp_ × (AR)(R)	production of ARR complex
α_bn_ = k_bn_ × (ARR)	degradation of ARR complex to AR and R

**Table 3:** Variable change matrix.

				Variable Change Matrix						
	*k_2p_*	*k_2n_*	*k_1p_*	*k_1n_*	*k_on_*	*k_off_*	*k_mp_*	*k_mn_*	*k_bp_*	*k_bn_*
(A_b_)	1	−1	−1	1	0	0	0	0	0	0
(A_c_)	0	0	1	−1	−1	1	0	0	0	0
(A_o_)	0	0	0	0	1	−1	−1	1	0	0
(AR)	0	0	0	0	0	0	1	−1	−1	1
(ARR)	0	0	0	0	0	0	0	0	1	−1
(R)	0	0	0	0	0	0	−1	1	−1	1

**Table 4:** Results of 25 calculations with constant initial value of (*R*) and fixed reaction constants.

	(AR) and (ARR) Similar			
	k_mp_ = 10^−4^	k_mn_ = 10^−6^	k_bp_ = 2 × 10^−6^	k_bn_ = 10^−8^
	Total (R)	(AR)	(ARR)	Total (A)
mean	100,000	38,350	30,825	69,174
SD	0	115	58	57
CV%	0	0.30	0.19	0.08

**Table 5:** Summary of model results for various reaction conditions and varying values of initial (*R*).

**(AR) and (ARR) Similar**				
	**k_mp_ = 10^−4^**	**k_mn_ = 10^−6^**	**k_bp_ = 2 × 10^−6^**	**k_bn_ = 10^−8^**
	Total (R)	(AR)	(ARR)	Total (A)
mean	100,808	38,328	31,240	69,568
SD	7000	314	3368	3632
CV%	6.9	0.61	8.86	5.2
**(AR) Dominates**				
	**k_mp_ = 2 × 10^−4^**	**k_mn_ = 10^−6^**	**k_bp_ = 10^−7^**	**k_bn_ = 10^−9^**
	Total (R)	(AR)	(ARR)	Total (A)
mean	99,747	90,696	4525	95,222
SD	6242	4803	725	5522
CV%	6.26	5.29	16.07	5.80
**(ARR) Dominates**				
	**k_mp_ = 10^−4^**	**k_mn_ = 10^−6^**	**k_bp_ = 2 × 10^−4^**	**k_bn_ = 10^−8^**
	Total (R)	(AR)	(ARR)	Total (A)
mean	98,223	1726	48,248	49,975
SD	7862	35	3931	3931
CV%	8.01	2.04	8.15	7.87

**Table 6:** Model calculation results for fluctuating bivalent binding.

(R) Fixed, k_bp_ Varies				
Reaction Constants	k_mp_ = 10^−4^	k_mn_ = 10^−6^	k_bp_ = 10^−7^ − 10^−6^	k_bn_ = 10^−9^
population	Total (R)	(AR)	(ARR)	Total (A)
mean	100,000	72,242	13,877	86,119
SD	0	19,120	9563	9558
CV%	0	26.5	68.9	11.1

**Table 7:** Model calculation results for fluctuating (*R*) and fluctuating k_bp_.

(R) Varies, k_bp_ Varies				
Reaction Constants	k_mp_ = 10^−4^	k_mn_ = 10^−6^	k_bp_ = 10^−6.5 ± 0.5^	k_bn_ = 10^−8^
population	Total (R)	(AR)	(ARR)	Total (A)
mean	100,522	73,725	13,399	87,124
SD	5240	18,045	9201	9587
CV%	5.2	24.5	68.7	11.0

## Abbreviations

MFI	mean fluorescence intensity
ABC	antibodies bound per cell
mAb	monoclonal antibody
CD4	cluster of differentiation 4
CV%	coefficient of variation
FITC	fluorescein isothiocyanate
APC	Allophycocyanin
(AR)	number of mAbs CD4 receptor complexes on T cell
(ARR)	number of mAb two CD4 receptor complexes on T cell
(R)	number of unbound CD4 receptors on the T cell
PBMC	peripheral blood mononuclear cell
PBS	phosphate buffer saline
FBS	fetal bovine serum
SD	Standard deviation

## References

[1] Wang L., Gaigalas A., Marti G., Hoffman R. (2008). Towards quantitative fluorescence measurements with multicolor flow cytometry. Cytometry.

[2] Zhu J., Paul W.E. (2008). CD4 T cells: Fates, functions, and faults. Blood.

[3] Dai X., Mei Y., Cai D., Han W. (2019). Standardizing CAR-T therapy: Getting it scaled up. Biotechnol. Adv..

[4] Wang L., Degheldy H., Abbasi F., Mostowski H., Marti G., Bauer S., Hoffman R., Gaigalas A. (2016). Quantitative flow cytometry measurements in antibodies bound per cell based on a CD4 reference. Curr. Protoc. Cytom..

[5] Wang M., Misakian M., He H.-J., Bajcsy P., Abbasi F., Davis J.M., Cole K.D., Turko I.V., Wang L. (2014). Quantifying CD4 receptor protein in two human CD4^+^lymphocyte preparations for quantitative flow cytometry. Clinic. Proteom..

[6] Protter P.E. (2005). Stochastic Integration and Differential Equations.

[7] Kearsley A.J., Gadhyan Y., Wallace W.E. (2014). Stochastic regression modeling of chemical spectra. Chemom. Intell. Lab. Syst..

[8] Gillespie D.T. (1977). Exact Stochastic Simulation of Coupled Chemical Reactions. J. Phys. Chem..

[9] Gillespie D.T. (2007). Stochastic Simulation of Chemical Reactions. Ann. Rev. Phys. Chem..

[10] Erban R., Chapman S.J., Maini P.K. (2007). A Practical Guide to Stochastic Simulations of Reaction-Diffusion Processes. arXiv.

[11] Pero J.K., Hass E.M., Thompson N.L. (2006). Size Dependence of Protein Diffusion Very Close to Membrane Surfaces: Measurement by Total Internal Reflection with Fluorescence Correlation Spectroscopy. J. Phys. Chem. B.

[12] Davis K.A., Abrams B., Iyer S., Hoffman R.A., Bishop J.E. (1998). Determination of CD4 Antigen Density on Cells. Role of Antibody Valency, Avidity, Clones, and Conjugation. Cytometry.

[13] Wang L., Gaigalas A.K., DeRose P.C. (2018). A Model for the Binding of Fluorescently Labeled Anti-Human CD4 Monoclonal Antibodies to CD4 Receptors on Human Lymphocytes. J. Res. NIST.

[14] Minton A.P. (2006). How can biochemical reactions within cells differ from those in a test tube?. J. Cell Sci..

[15] Malaspina D.C., Longo G., Szleifer I. (2017). Behavior of ligand binding assays with crowded surfaces: Molecular model of antigen capture by antibody-conjugated nanoparticles. PLoS ONE.

[16] Care B.R., Soula H.A. (2011). Impact of receptor clustering on ligand binding. BMC Syst. Biol..

[17] Du X., Xia Y.-L., Ai S.-M., Liang J., Sang P., Ji X.-L., Liu S.-Q. (2016). Insights into Protein-Ligand Interactions: Mechanisms, Models, and Methods. Int. J. Mol. Sci..

[18] Dahanayake J.N., Mitchell-Kock K.R. (2018). How Does Solvation Layer Mobility Affect Protein Structural Dynamics?. Front. Mol. Biosci..

[19] Ebbinghaus S., Kim S.J., Heyden M., Yu X., Heugen U., Gruebele M., Leitner D.M., Havenith M. (2007). An extended dynamical hydration shell around proteins. Proc. Natl. Acad. Sci. USA.

[20] Bizzarri A.R., Vegh A.G., Varo G., Cannistraro S. (2019). Interaction Force Fluctuations in Antigen-Antibody Biorecognition Studies by Atomic Force Spectroscopy. ACS Omega.

[21] Meyer-Olson D., Brady K.W., Bartman M.T., O’Sullivan K.M., Simons B.C., Conrad J.A., Duncan S.L., Siddique A., Draenert R., Addo M. (2006). Fluctuations of functionally distinct CD8^+^ T-cell clonotypes demonstrate flexibility of the HIV-specific TCR repertoire. Blood.

[22] Levine B.L., Miskin J., Wonnacott K., Keir C. (2017). Global Manufacturing of CAR T Cell Therapy. Mol. Ther. Methods Clin. Dev..

